# Maternal anxiety, depression and asthma and adverse pregnancy outcomes – a population based study

**DOI:** 10.1038/s41598-019-49508-z

**Published:** 2019-09-11

**Authors:** Gustaf Rejnö, Cecilia Lundholm, Sara Öberg, Paul Lichtenstein, Henrik Larsson, Brian D’Onofrio, Kjell Larsson, Sissel Saltvedt, Bronwyn K. Brew, Catarina Almqvist

**Affiliations:** 1 Department of Medical Epidemiology and Biostatistics, Karolinska Institutet, Stockholm, Sweden; 2 Obstetrics and Gynaecology Unit, Söderjukhuset, Stockholm, Sweden; 3 School of Medical Sciences, Örebro Universitet, Örebro, Sweden; 4 Department of Psychological and Brain Sciences, Indiana University, Bloomington, USA; 5 Institute of Environmental Medicine, Karolinska Institutet, Stockholm, Sweden; 6 Department of Women’s and Children’s Health, Karolinska Institutet, Stockholm, Sweden; 7 Obstetrics & Gynaecology Unit, Karolinska University Hospital, Stockholm, Sweden; 8 Pediatric Allergy and Pulmonology Unit at Astrid Lindgren Children’s Hospital, Karolinska University Hospital, Stockholm, Sweden

**Keywords:** Anxiety, Depression, Asthma, Epidemiology, Risk factors

## Abstract

To evaluate associations between maternal anxiety or depression and adverse pregnancy outcomes, taking possible familial confounding and interaction with asthma into account, we conducted a cohort study of all singleton births in Sweden 2001–2013. We retrieved information about pregnancy, diagnoses of anxiety/depression, asthma, and prescribed medication from the Swedish Medical Birth, National Patient, and Prescribed Drug Registers. We estimated associations with regression models, performed cousin and sibling comparisons, and calculated interactions. In 950 301 identified pregnancies; 5.9% had anxiety/depression and 4.0% had asthma. Anxiety/depression was associated with adverse pregnancy outcomes (e.g. preeclampsia, adjusted Odds Ratio 1.17 (95% Confidence Interval 1.12, 1.22), instrumental delivery (1.14 (1.10, 1.18)), elective (1.62 (1.57, 1.68)) and emergency (1.32 (1.28, 1.35)) caesarean section (CS)). Their children had lower birth weight (−54 g (−59, −49)) and shorter gestational age (−0.29 weeks (−0.31, −0.28)). Associations were not confounded by familial factors and asthma did not modify the effect of anxiety/depression for outcomes other than elective CS, p < 0.001. In women with anxiety/depression diagnosis, untreated women had higher odds of elective CS compared to women on medication (1.30 (1.17, 1.43)). In conclusion, anxiety/depression should be considered when evaluating pregnant women’s risk of complications such as preeclampsia and non-vaginal deliveries.

## Introduction

Anxiety and depression are common disorders that affect a large part of the population^1^, and studies have indicated that the prevalence of depression is as high as 6–15% in pregnant women^2,3^. Anxiety and depression are closely associated conditions that may affect not only psychological well-being but also the prognosis of other diseases^4,5^, and depression or anxiety is associated with asthma diagnosis^6^. Previous studies have also shown that maternal psychological stress (anxiety and/or depression) is associated with adverse outcomes during pregnancy, as well as adverse events in the child^7–10^.

The mechanisms for the association between anxiety or depression and adverse outcomes in pregnancy are yet to be clarified. Although generally recommended during pregnancy, antidepressant medication, such as selective serotonin re-uptake inhibitors (SSRIs), are not always used by the pregnant woman, perhaps out of concern for adverse foetal effects associated with the medication^11^. However, previously observed associations between SSRIs and low birth weight or preterm birth may be confounded by factors such as the depressive disorder itself or specific characteristics of women taking SSRI during pregnancy^12,13^. In addition, common familial factors (such as shared genes and environment) may be confounders for the association. In order to adjust for those factors a family-design approach can be used^14^, allowing adjustment for all familial factors that cousins and siblings share. Previous studies have also investigated whether familial factors may confound the association between maternal anxiety and depression during pregnancy and offspring asthma using cousin analyses and found that the association between maternal anxiety or depression and offspring asthma was not due to familial confounding^15^.

In earlier studies, we have assessed the association between maternal asthma and adverse pregnancy-, delivery- and perinatal outcomes^16,17^. There are known associations between maternal prenatal anxiety or depression and maternal or offspring asthma^6,15,18–20^ as well as between maternal anxiety or depression and adverse pregnancy outcomes similar to outcomes seen in asthmatic mothers^7,10,21,22^, perhaps due to increased levels of stress hormones^23^. Thus, the presence of both conditions may increase the risk of adverse pregnancy outcomes above what would be expected from the risk of each condition alone. To our knowledge such an interaction has not been previously assessed and could help determine which subgroups may benefit the most from an intervention, as well as help to understand the mechanisms leading to adverse pregnancy, delivery, and birth outcomes.

Our aim was to study pregnancy outcomes in women with maternal prenatal anxiety or depression, and to determine if potential associations were confounded by factors shared by cousins and siblings respectively. We also aimed to examine possible interaction (additive or multiplicative^24^) between maternal anxiety or depression and maternal asthma on the risk of adverse pregnancy outcomes.

## Results

In the study population of 661 956 women we identified a total of 1 075 153 pregnancies during the study period from 2001 to 2013. Individuals with missing covariates (124 852) were excluded so the final cohort included 950 301 pregnancies by 616 667 mothers. After random selection of one pregnancy per individual for the cousin and sibling analyses, we identified 35 156 women discordant for maternal anxiety and depression in 16 175 cousin clusters and 17 638 women in 8145 sister clusters giving birth during the study period. In the final cohort, the prevalence of prenatal maternal anxiety or depression according to our definition was 5.9% (56 297), and 4.0% (37 686) fulfilled our criteria for prenatal maternal asthma. In total 4892 (0.5%) had both anxiety/depression and asthma during the study period. The women in this subgroup were less educated, had a slightly higher BMI, and less likely to live with the child’s father the year prior to delivery compared to the whole cohort, Table 1.

**Table 1:** Characteristics of the study population according to current asthma* and/or anxiety/depression^†^ status.

	Whole population	All with anxiety/depression	All with asthma	All with anxiety/depression and asthma
	N = 950 301, n (%)	N = 56 297, n (%)	N = 37 686, n (%)	N = 4892, n (%)
**Maternal Characteristics**				
*Age*				
*Median age*	30	30	31	31
*Mean*	30.2	30.2	30.6	30.7
*≤19*	13 146 (1.4)	1147 (2.0)	568 (1.5)	93 (1.9)
*20–24*	117 688 (12.4)	8257 (14.7)	4552 (12.1)	668 (13.7)
*25–29*	288 170 (30.3)	15 547 (27.6)	10 485 (27.8)	1270 (26.0)
*30–34*	339 652 (35.7)	18 011 (32.0)	12 900 (34.2)	1549 (31.7)
*>34*	191 645 (20.2)	13 335 (23.7)	9181 (24.4)	1312 (26.8)
***Early pregnancy BMI***				
*Median*	24	24	24	25
*Mean*	24.6	25.3	25.7	26.6
*<18,5*	20 289 (2.1)	1401 (2.5)	744 (2.0)	96 (2.0)
*18,5–24,9*	586 484 (61.7)	31 008 (55.1)	19 853 (52.7)	2283 (46.7)
*25–29,9*	232 951 (24.5)	14 668 (26.1)	10 201 (27.1)	1319 (27.0)
*≥30*	110 577 (11.6)	9220 (16.4)	6888 (18.3)	1194 (24.4)
***Parity***				
*1*	434 380 (45.7)	27 574 (49.0)	18 330 (48.6)	2410 (49.3)
*2–3*	475 819 (50.1)	25 095 (44.6)	17 590 (46.7)	2105 (43.0)
*≥4*	40 102 (4.2)	3628 (6.4)	1766 (4.7)	377 (7.7)
***Cigarettes smoked/day***				
*0*	876 929 (92.3)	47 141 (83.7)	34 319 (91.1)	3969 (81.1)
*1–9*	55 273 (5.8)	6340 (11.3)	2441 (6.5)	611 (12.5)
*>9*	18 099 (1.9)	2816 (5.0)	926 (2.5)	312 (6.4)
***Country of birth***				
*Sweden*	904 040 (95.1)	53 778 (95.5)	36 289 (96.3)	4706 (96.2)
*Denmark, Norway, Finland, Iceland*	5322 (0.6)	348 (0.6)	192 (0.5)	33 (0.7)
*Other countries*	40 939 (4.3)	2171 (3.9)	1205 (3.2)	153 (3.1)
***Cohabitation/marital status***				
*Living with baby’s father, married*	354 344 (37.3)	17 223 (30.6)	14 077 (37.4)	1522 (31.1)
*Living with baby’s father, unmarried*	548 776 (57.7)	32 126 (57.1)	21 121 (56.0)	2675 (54.7)
*Not living with baby’s father*	47 181 (5.0)	6948 (12.3)	2488 (6.6)	695 (14.2)
***Highest attained education level***				
*≤9 years*	82 869 (8.7)	9819 (17.4)	3807 (10.1)	920 (18.8)
*10–12 years*	411 578 (43.3)	24 715 (43.9)	15 121 (40.1)	2104 (43.0)
*≥13 years*	455 854 (48.0)	21 763 (38.7)	18 758 (49.8)	1868 (38.2)

*Maternal asthma during the year before pregnancy up until delivery, identified as having at least two dispenses of asthma medication in the Swedish Prescribed Drug Register, or asthma diagnosis during in the Swedish National Patient Register during the same period. ^†^Antidepressant medication or anxiety/depression diagnoses recorded in the Swedish Medical Birth Register, anxiety/depression diagnosis in the Swedish National Patient Register, and/or antidepressant medication dispensed at least twice in the year before pregnancy until birth according to the Swedish Prescribed Drug Register.

### Main analysis

Maternal anxiety or depression was associated with higher odds for preeclampsia or eclampsia, adjusted odds ratio, aOR, 1.17 (95% confidence interval, CI, 1.12, 1.22) but not with placental abruption, Table 2. For instrumental vaginal delivery, elective and emergency caesarean section the odds were also higher among women with anxiety or depression compared to women with no anxiety or depression (e.g. elective caesarean section aOR 1.62 (95% CI 1.57, 1.68).

**Table 2:** The association between anxiety or depression during pregnancy* and pregnancy and labor outcomes assessed with logistic and linear regression; presenting crude and adjusted models in the full cohort followed by comparison of maternal cousins and sisters.

	Total population	Current anxiety/depression	Full cohort comparison		Cousin comparison		Sister comparison	
	N = 950 301	n = 56 297	Crude	Adjusted^†^	Double discordant clusters	Adjusted^†^	Double discordant clusters	Adjusted^†^
	n(%)	n(%)	OR or Beta (95% CI)	OR or Beta (95% CI)	Total individuals/clusters 286 780/128 602	OR or Beta (95% CI)	Total individuals/clusters 158 833/75 391	OR or Beta (95% CI)
**Pregnancy Characteristics**								
*Preeclampsia or Eclampsia*	38 727 (4.1)	2771 (4.9)	1.24 (1.19, 1.29)	1.17 (1.12, 1.22)	1721	1.14 (1.02, 1.26)	728	1.10 (0.88, 1.32)
*Placental abruption*	3340 (0.4)	231 (0.4)	1.18 (1.03, 1.35)	1.04 (0.90, 1.19)	145	1.24 (0.67, 1.81)	56	0.99 (0.30, 1.69)
**Labor Characteristics**								
*Mode of Delivery*								
*Vaginal non-instrumental delivery*	723 884 (76.2)	39 673 (70.5)	Ref.	Ref.	Ref.	Ref.	Ref.	Ref.
*Elective CS (before start of labor)*	59 868 (6.3)	5349 (9.5)	1.69 (1.64, 1.75)	1.62 (1.57, 1.68)	2520	1.72 (1.48, 1.96)	1148	1.63 (1.29, 1.96)
*Vaginal instrumental delivery*	71 004 (7.5)	4279 (7.6)	1.11 (1.07, 1.14)	1.14 (1.10, 1.18)	2877	1.17 (1.04, 1.30)	1199	1.22 (1.00, 1.44)
*Emergency CS prior to or after start of labor*	86 811 (9.1)	6510 (11.6)	1.40 (1.36, 1.44)	1.32 (1.28, 1.35)	3509	1.38 (1.26, 1.50)	1610	1.38 (1.15, 1.62)
*Missing*	8734 (0.9)	486 (0.9)						

*Maternal anxiety and/or depression from the year before pregnancy until delivery. ^†^Adjusted for country of birth, smoking, cohabitation, maternal education level, BMI, age.

Mean birth weight was lower (−54 g (95% CI −59, −49)) and gestational age shorter (−0.29 weeks (95% CI −0.31, −0.28)) in the maternal anxiety or depression group compared to the general population, while the odds of delivering a child small for gestational age (aOR 1.03 (95% CI 0.97, 1.09)) did not differ. In contrast, the odds of large for gestational age were slightly higher among women with anxiety or depression (aOR 1.09 (95% CI 1.04, 1.13)) compared to the general population, Table 3.

**Table 3:** The association between anxiety or depression during pregnancy* and birth outcomes assessed with logistic and linear regression; presenting crude and adjusted models in the full cohort followed by comparison of maternal cousins and sisters.

	Total population	Current anxiety/depression	Full cohort comparison		Cousin comparison		Sister comparison	
	N = 950 301	n = 56 297	Crude	Adjusted^†^	Double discordant clusters	Adjusted^†^	Double discordant clusters	Adjusted^†^
	n(%)	n(%)	OR or Beta (95% CI)	OR or Beta (95% CI)	Total individuals/clusters 286 780/128 602	OR or Beta (95% CI)	Total individuals/clusters 158 833/75 391	OR or Beta (95% CI)
**Birth outcomes**								
*Sex of offspring*								
*Boy*	488 504 (51.4)	28 947 (51.4)						
*Girl*	461 797 (48.6)	27 350 (48.6)						
*Missing*								
*Birth weight, mean grams*	3571	3509	−66 (−71, −61)^‡^	−54 (−59, −49)^‡^	16,168	−57 (−67, −48)^‡^	8144	−58 (−74, −43)^‡^
*Missing*	1839 (0.2)	101 (0.2)						
*Gest. age, mean weeks*	39.4	39.1	−0.32 (−0.34, −0.31)^‡^	−0.29 (−0.31, −0.28)^‡^	16,169	−0.28 (−0.32, −0.23)^‡^	8145	−0.25 (−0.31, −0.19)^‡^
*Missing*	0 (0.0)	0 (0.0)						
*Small for gestational age*	19 279 (2.0)	1346 (2.4)	1.20 (1.13, 1.27)	1.03 (0.97, 1.09)	830	1.03 (0.86, 1.21)	424	1.14 (0.76, 1.51)
*Missing*	1871 (0.2)	106 (0.2)						
*Large for gestational age*	36 224 (3.8)	2449 (4.4)	1.16 (1.11, 1.21)	1.09 (1.04, 1.14)	1398	1.19 (1.02, 1.36)	617	1.07 (0.79, 1.34)
*Missing*	1871 (0.2)	106 (0.2)						

*Maternal anxiety and/or depression from the year before pregnancy until delivery. ^†^Adjusted for country of birth, smoking, cohabitation, maternal education level, BMI, age. ^‡^P < 0.001.

Adjusting for familial factors shared by first cousins and sisters generally produced small changes to point estimates but wider confidence intervals. In sisters, for instance, the adjusted odds ratio for preeclampsia or eclampsia was 1.10 (95% CI 0.88, 1.32), Table 2. Similar patterns could be seen for the perinatal outcomes in Table 3.

### Interaction analyses

Next, we evaluated whether maternal asthma interacted with anxiety or depression on the risk of adverse pregnancy outcomes, and found that the combination of anxiety or depression and asthma showed the highest odds ratios for most of our studied outcomes compared to the reference group having neither exposure, Table 4. In the case of preeclampsia or eclampsia for instance, women with both anxiety/depression and asthma had an aOR of 1.45 (95% CI 1.29, 1.64), while the corresponding OR was 1.15 (95% CI 1.10, 1.20) for women with anxiety/depression but no asthma, and 1.28 (95% CI 1.22, 1.35) for women without anxiety/depression but with asthma. Despite these findings, there were no strong indications of additive interaction (relative excess risk due to interaction) being present, for example preeclampsia or eclampsia RERI 0.02 (95% CI −0.16, 0.20). Similarly, there were no signs of additive interaction for the continuous outcomes of birth weight and gestational age (for example birth weight −17 grams (95% CI −35, 2 grams), gestational age −0.05 weeks (95% CI −0.11, 0.13 weeks).

**Table 4:** Additive Interaction between maternal anxiety/depression and maternal asthma* for the effect on adverse pregnancy, delivery and perinatal outcomes. Each group is compared to the no anxiety/depression and no asthma group.

	Neither anxiety/depression nor asthma		Anxiety/depression without asthma		Asthma without anxiety/depression		Anxiety/depression with asthma		Additive interaction^†^
	n = 861 210		n = 51 405		n = 32 794		n = 4892		For adjusted estimates
	n(%)	Reference	n(%)	OR or Beta (95% CI)^‡^	n(%)	OR or Beta (95% CI)^‡^	n(%)	OR or Beta (95% CI)^‡^	Adjusted^‡^
**Pregnancy Characteristics**									
*Preeclampsia or Eclampsia*	34 135 (4.0)	1.00	2446 (4.8)	1.15 (1.10, 1.20)	1821 (5.6)	1.28 (1.22, 1.35)	325 (6.6)	1.45 (1.29, 1.64)	0.02 (−0.16, 0.20)
*Placental abruption*	2962 (0.3)	1.00	209 (0.4)	1.04 (0.90, 1.20)	147 (0.5)	1.29 (1.09, 1.52)	22 (0.5)	1.10 (0.72, 1.68)	−0.23 (−0.77, 0.30)
**Labor Characteristics**									
*Mode of Delivery*									
*Vaginal non-instrumental delivery*	660 991 (77.5)	1.00	36 465 (70.9)	Ref.	23 220 (70.8)	Ref.	3208 (65.6)	Ref.	Ref.
*Elective CS (before start of labor)*	51 628 (6.1)	1.00	4789 (9.3)	1.62 (1.57, 1.68)	2891 (8.8)	1.49 (1.43, 1.55)	560 (11.5)	2.01 (1.82, 2.21)	−0.10 (−0.30, 0.10)
*Vaginal instrumental delivery*	64 141 (7.5)	1.00	3903 (7.6)	1.14 (1.10, 1.18)	2584 (7.9)	1.15 (1.10, 1.20)	376 (7.7)	1.27 (1.14, 1.41)	−0.02 (−0.17, 0.12)
*Emergency CS prior to or after start of labor*	76 420 (8.9)	1.00	5791 (11.3)	1.30 (1.26, 1.34)	3881 (11.8)	1.34 (1.29, 1.39)	719 (14.7)	1.68 (1.54, 1.82)	0.03 (−0.12, 0.17)
*Missing*	8030 (0.9)		457 (0.9)		218 (0.7)		29 (0.6)		
**Birth outcome and post-partum**									
*Birth weight, mean grams*	3577	3577	3515	−49 (−55, −44)^§^	3523	−74 (−81, −68)^§^	3443	−140 (−157, −123)^§^	−17 (−35, 2)
*Missing*	1677 (0.2)		92 (0.2)		61 (0.2)		9 (0.2)		
*Gest. age, mean weeks*	39.4	39.4	39.1	−0.28 (−0.30, −0.26)^§^	39.3	−0.14 (−0.17, −0.12)^§^	38.9	−0.48 (−0.53, −0.42)^§^	−0.05 (−0.11, 0.13)
*Missing*	0 (0.0)		0 (0.0)		0 (0.0)		0 (0.0)		
*Small for gestational age*	17 045 (2.0)	1.00	1180 (2.3)	1.00 (0.94, 1.07)	888 (2.7)	1.40 (1.31, 1.50)	166 (3.4)	1.47 (1.26, 1.72)	0.07 (−0.19, 0.33)
*Missing*	1703 (0.2)		97 (0.2)		62 (0.2)		9 (0.2)		
*Large for gestational age*	32 559 (3.8)	1.00	2239 (4.4)	1.10 (1.05, 1.15)	1216 (3.7)	0.84 (0.79, 0.89)	210 (4.3)	0.90 (0.78, 1.04)	−0.04 (−0.18, 0.11)
*Missing*	1703 (0.2)		97 (0.2)		62 (0.2)		9 (0.2)		

*Anxiety, depression and asthma from the year before pregnancy until end of pregnancy. ^†^Additive interaction exists if not equal to zero. ^‡^Adjusted for country of birth, smoking, cohabitation, maternal education level, BMI, age. ^§^P < 0.01.

**Figure 1:**
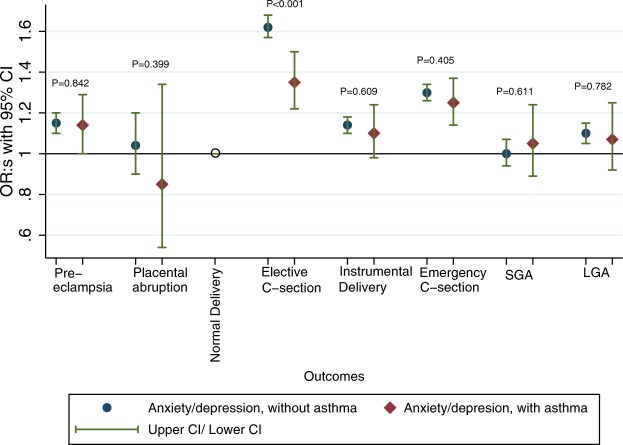
Adjusted odds ratios for the association between anxiety or depression and adverse pregnancy, delivery and perinatal outcomes in all women without asthma and all women with asthma respectively, from the year before pregnancy until delivery. P-values are from testing a multiplicative interaction between anxiety or depression and asthma.

**Figure 2:**
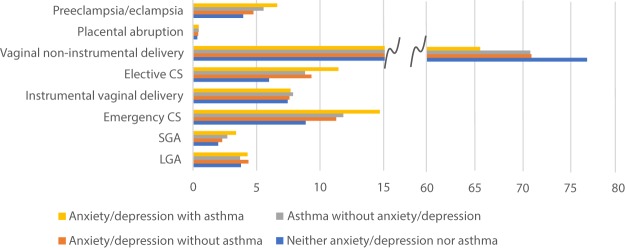
Women in each group of exposures and frequencies of outcomes. Frequencies shown as percentages.

We also assessed multiplicative interaction. Among women with maternal anxiety or depression, asthma status made little difference on the effect on pregnancy, labour and birth outcomes (aOR for preeclampsia or eclampsia without asthma 1.15 (95% CI 1.10, 1.20) and with asthma 1.14 (95% CI 1.00, 1.29), Fig. 1 and Table S1. Frequencies of pregnant women with the various outcomes in the different groups are shown in Fig. 2. When assessing potential interaction between anxiety or depression and asthma on the multiplicative scale, it was only the effect on elective caesarean section that turned out altered, and the positive association between anxiety or depression and elective caesarean section was less pronounced in women with asthma than in women without (p-value < 0.001). Figure 1 and Table S1.

### Sensitivity analyses

There were no major differences in the estimates for birth years 2001–2005 and 2010–2013, Table S2.

In the analysis of women with a diagnosis of maternal anxiety or depression contrasting those with or without medication, those on medication did not have any increased risk of pregnancy complications. On the contrary, women without medication had an increased risk of elective caesarean section aOR 1.30 (95% CI 1.17, 1.43), Table S3.

## Discussion

In this population-based cohort study of more than one million Swedish births by 661 956 women in the years 2001 to 2013, we found that maternal prenatal anxiety or depression during pregnancy increases the odds of adverse pregnancy outcomes, such as preeclampsia or eclampsia, emergency caesarean section, low gestational age, low birth weight and large for gestational age. The robustness of estimates in cousin and sibling analyses compared to the full cohort analysis indicates that the associations are not confounded by genetic or environmental factors shared within families.

We also found multiplicative interaction of the effects of maternal asthma and prenatal anxiety or depression on the risk of elective caesarean section. However, there was no significant additive interactions between maternal asthma and maternal prenatal anxiety or depression for this or any other outcomes. The general lack of both additive and multiplicative interaction, elective caesarean section excluded, is a bit surprising but might be explained by the high degree of comorbidity of these diseases^25,26^ and confirms that it is important to address both asthma and mental health issues in pregnant women.

Previous studies have examined the association between anxiety or depression and hypertensive disorders in pregnancy^22,27^ with similar results as we have seen in our study. In a retrospective study of 22 825 women, emergency caesarean section was shown to be associated with anxiety or depression^28^. Gestational age and birth weight in association with anxiety or depression has also been assessed previously and finding suggest effects on at least the former outcomes^29^. Using another type of design, Viktorin^12^ and Sujan^13^ compared gestational outcomes of mothers who were discordant for SSRI and other anti-depressive medication during two pregnancies and concluded that exposure to anti-depressive medication may be causally related to preterm birth. In our analyses we extended this to additional diagnoses and medication, and in a similar way adjusted for familial confounding. We saw an association with low birth weight in both the full cohort and in the cousin and sibling sub-groups, however we couldn’t see any association with the child being small for gestational age, which is similar to the results by Sujan *et al*.

We have previously studied the association between maternal asthma and adverse pregnancy outcomes, such as preeclampsia, instrumental delivery, caesarean section, and child being small for gestational age^16,17^, and those findings were further confirmed in this study.

In sensitivity analysis of women with a diagnosis of prenatal anxiety or depression with or without medication, we found slightly lower point estimates in the group without medication for most outcomes except elective caesarean section, although the inability to detect differences in estimates between the groups also could be explained by loss of power. Those without medication could for different reasons be more reluctant to use medication during pregnancy^30^, maybe due to higher levels of anxiety for the pregnancy and foetus. A possible explanation for the elective caesarean section to stand out both in the sensitivity analysis of women with or without medication and in the multiplicative interaction analysis is that there is something intrinsically different about this outcome. The other outcomes are either measurable (such as gestational age or birth weight), based on specific criteria (pre-eclampsia^31^) or occur after specific pre-established conditions have been met (for instance instrumental delivery or emergency caesarean section when there is sign of foetal distress). The indication for elective caesarean section is much wider and more heterogeneous, and although in Sweden still mostly done for valid medical reasons, there is an increase of elective caesarean section on wider indications^32^. If a women has untreated anxiety or depression the obstetrician might be more open to elective caesarean section on a wider indication, e.g. the perception that the woman may be less capable of going through normal labour.

This is the first study to examine maternal anxiety or depression and the association with several clinically important pregnancy-, delivery- and perinatal outcomes, taking familial confounding into account. The study has several major strengths. It is a large population-based study with close to a million singleton births and 16 175 clusters of cousin pregnancies and 8145 clusters of sister pregnancies discordant for maternal anxiety or depression. The family design allowed us to adjust for unmeasured confounders such as shared genes and common environment. The use of prospectively and independently collected data has eliminated the risk for recall bias. The size of the population has also enabled us to adjust for many known confounders. It is also the first study to examine how additive and multiplicative interaction between maternal asthma and anxiety or depression affects the studied outcomes. Previous studies have assessed the association between the two^15,20^ but not addressed whether there is a mutual effect on pregnancy outcomes in order to evaluate clinical relevance and possible etiology. Furthermore, we have looked at anxiety or depression with or without medication in order to determine if the observed associations were driven by medication use.

There are also limitations to the study. The use of family design has limitations^33^ that are important to consider, for instance the influence of measurement errors and unmeasured confounding may be inflated from the selection of discordant family members. However, by collecting exposure data from different and independent sources measurement error is likely to be reduced. Additionally, there might be confounders that are shared to a less extent within families. This would lead to higher estimates in the within-analyses and might be counteracted by measurement errors. There is also a large drop of power in the family-design analyses since the only informative participants are those discordant for anxiety or depression and the outcome. This can be a reason for the statistically non-significant results across many of the outcomes. Also, because the diagnoses collected from the Patient Register for this study only include specialist care diagnoses, we are missing those diagnosed by general practitioners, who handle a large part of asthma, anxiety and depression patients in Sweden. The more severe cases are however more likely to have a diagnosis in the register (and thus found in our searches). For instance, the prevalence of asthma was lower than expected and at least for the earlier part of the cohort we suspect that many of the asthma diagnoses belong to women with a more severe disease. Sensitivity analyses however showed similar results for the earlier and later birth years. We have used diagnosis and medication for anxiety and depression as a measure of psychological stress, although medications for depression are also used in other conditions, such as chronic pain management^34^; still, such conditions are also stressful and known to have high co-morbidity with depression and anxiety^35^. Also, because this is a register-based study we can only speculate the reasons for choosing elective caesarean section.

In conclusion, we show that there is an association between maternal prenatal anxiety or depression and adverse pregnancy outcomes, which is not confounded by familial factors shared by cousins and siblings. We also show that there is no interaction between maternal anxiety or depression and maternal asthma for any of the outcomes studied, except for elective caesarean section. Clinically, these risks must be highlighted and especially the higher occurrence of elective caesarean section addressed. Further research should examine how well-controlled asthma as well as optimally treated anxiety or depression may affect the rates of caesarean section and other outcomes.

## Methods

### Study design and population

To study the effects of maternal anxiety or depression during pregnancy, and possible interaction with maternal asthma, on the risk of adverse pregnancy and delivery outcomes we used a cohort consisting of all Swedish births between 2001 and 2013. The Medical Birth Register^36^ was used to identify women giving birth during the study period. Using the Swedish Personal Identity Number, a unique identifier for each permanent resident in Sweden^37^, we then linked the cohort to the Swedish Prescribed Drug Register^38^ and the Patient Register^39^.

Using the Multi Generation Register^40^ we identified cousins and sisters from the same family giving birth during the study period.

Multiple gestation pregnancies were excluded because of characteristics that differ substantially from singleton pregnancies, in order to facilitate interpretation of results^41^.

### Exposure

Our exposures were maternal anxiety and/or depression from the year before pregnancy until delivery, identified in the inpatient and/or outpatient part of the Patient register as having ICD-10 diagnosis codes F30–34, F38-42, F44-45 or F48 – and in the Swedish Prescribed Drug Register as ATC-codes N05B and N06A – dispensed at least twice during the year before pregnancy up until delivery^15^.

Our second exposure was maternal asthma during the year before pregnancy up until delivery, identified as having at least two dispenses of asthma medication (ATC codes R03AC, R03AK, R03BA, R03DC) or asthma diagnosis (ICD-10 codes J45 and J46)^42^.

### Outcomes

Data on complications during pregnancy (pre-eclampsia, placental abruption), delivery (instrumental vaginal delivery, caesarean section) and birth outcomes (birth weight, gestational age, small- and large for gestational age^43^) were collected from the Medical Birth Register.

### Covariates

We selected possible confounders based on subject-matter informed directed acyclic graphs^44^, and collected data on these from the Medical Birth Register and the longitudinal integration database for health insurance and labor market studies (LISA by Swedish acronym) maintained by Statistics Sweden^45^. The covariates that were deemed possible confounders were self-reported maternal smoking and body mass index at the first antenatal visit, civil status, country of birth and socioeconomic status defined as the highest attained level of education.

### Statistical methods

All analyses were done using Stata 15^46^.

In a first step we estimated prevalence, crude and adjusted odds ratios and beta coefficients with 95% confidence intervals, using logistic and linear regression, for the selected outcomes using maternal anxiety or depression as exposure in the full cohort. To account for the clustering of observations within women with multiple deliveries, a sandwich estimator for the standard errors was used. In order to adjust for familial factors (genes and environment) shared by first cousins/sisters we then identified groups of cousins and full sisters discordant for the exposure and outcome, and randomly selected one pregnancy per individual. We used conditional logistic regression for the binary outcomes, conditioning on first cousins and full sisters (same mother and father) among the women giving birth during the study period, and for continuous outcomes a linear regression model with fixed effects estimator was used^47^.

Second, we included an interaction term between maternal anxiety or depression and maternal asthma to test for effect measure modification on the additive scale in the linear regression models (continuous outcomes) and the multiplicative scale in logistic regression (dichotomous outcomes). Differences in effects were tested with likelihood ratio test. Estimation of RERI (Relative Excess Risk due to Interaction), using the STATA package ic^48^, allowed us to evaluate potential interaction on the additive scale also for dichotomous outcomes.

Because data on medication is only available in the Swedish Prescribed Drug Register from 2006, the first half of the study population (2001–2005) may differ from the later (2006–2013) in that exposure classification was based on diagnosis in the former and diagnosis *or* medication in the latter. In order to discern possible differences between birth cohorts a sensitivity analysis was done, estimating odds ratios and beta coefficients for the association between anxiety or depression and the outcomes stratifying the cohort to either birth years 2001–2004 or 2010–2013. To assess the possibility that medication use could indicate a more severe case of anxiety or depression, a sensitivity analysis was also done among the women with a diagnosis of anxiety or depression and information on medication (2006 to 2013). Odds ratios and beta coefficients for the outcomes were estimated in women with and without medication for anxiety and depression.

### Ethical approval

Permission for this study was obtained from the Regional Ethical Review board in Stockholm, Sweden (reference 2013/862-31/5). In accordance with their decision, we did not obtain informed consent from participants involved in the study. All data were de-identified prior to analyses.

## Supplementary information

Interaction and sensitivity analyses

## Data Availability

Original data is held by Swedish National Board of Health and Welfare and Statistics Sweden and because of Swedish data storage laws we cannot make the data publicly available. However, any researcher can access the data by obtaining an ethical approval from a regional ethical review board and thereafter asking the Swedish National Board of Health and Welfare and Statistics Sweden for the original data.
