# Supplementary material for: Maternal anxiety, depression and asthma and adverse pregnancy outcomes – a population based study
Source: Sci Rep. 2019 Sep 11;9:13101. doi: 10.1038/s41598-019-49508-z (PMC6739415; doi:10.1038/s41598-019-49508-z)
Supplement: Supplementary file 1 — Interaction and sensitivity analyses [file 41598_2019_49508_MOESM1_ESM.pdf]

Maternal anxiety, depression and asthma and adverse pregnancy outcomes – a population based study

*Gustaf Rejnö[1, 2], MD PhD

Cecilia Lundholm[1], MSc

Sara Öberg[1], MD PhD

Paul Lichtenstein[1], PhD

Henrik Larsson[1, 3], PhD

Brian D'Onofrio[1, 4], PhD

Kjell Larsson[5], MD PhD

Sissel Saltvedt[6, 7], MD PhD

Bronwyn K Brew[1], PhD

Catarina Almqvist[1, 8], MD PhD

[1]Dept of Medical Epidemiology and Biostatistics, Karolinska Institutet, Stockholm, Sweden

[2]Obstetrics and Gynaecology Unit, Söderjukhuset, Stockholm, Sweden

[3]School of Medical Sciences, Örebro Universitet, Örebro, Sweden

[4]Department of Psychological and Brain Sciences, Indiana University, Bloomington, USA

[5]Institute of Environmental Medicine, Karolinska Institutet, Stockholm, Sweden

[6]Department of Women's and Children's Health, Karolinska Institutet, Stockholm, Sweden

[7]Obstetrics & Gynaecology Unit, Karolinska University Hospital, Stockholm, Sweden

[8]Pediatric Allergy and Pulmonology Unit at Astrid Lindgren Children's Hospital, Karolinska University Hospital, Stockholm, Sweden

Corresponding author:

Gustaf Rejnö, MD PhD

Department of Medical Epidemiology and Biostatistics

PO Box 281, Karolinska Institutet

SE 171 77 Stockholm, SWEDEN

Tel: +46 70 580 17 29

Fax: +46 8 31 49 75

E-mail: gustaf.rejno@ki.se

Table S1 Estimation of odds ratios and beta coefficients in women with anxiety/depression versus women without anxiety/depression, depending on asthma status*. Multiplicative interaction between maternal anxiety or depression and maternal asthma for the effect on pregnancy, delivery, and perinatal outcomes.

	All women without asthma		All women with asthma		Multiplicative interaction
	**Crude**	**Adjusted†**	**Crude**	**Adjusted†**	**for adjusted† estimates**
	**OR or Beta (95% CI)**	**OR or Beta (95% CI)**	**OR or Beta (95% CI)**	**OR or Beta (95% CI)**	**P-values**
**Pregnancy Characteristics**					
Preeclampsia or Eclampsia	1.21 (1.16 - 1.26)	1.15 (1.10 - 1.20)	1.21 (1.07 - 1.37)	1.14 (1.00 - 1.29)	0.842
Placental abruption	1.18 (1.03 - 1.36)	1.04 (0.90 - 1.20)	1.00 (0.64 - 1.57)	0.86 (0.55 - 1.34)	0.399
**Labor Characteristics**					
Mode of Delivery					
Vaginal non-instrumental delivery	Ref.	Ref.	Ref.	Ref.	Ref.
Elective CS (before start of labor)	1.68 (1.63 - 1.74)	1.62 (1.57 - 1.68)	1.40 (1.27 - 1.55)	1.35 (1.22 - 1.50)	<0.001‡
Vaginal instrumental delivery	1.10 (1.07 - 1.14)	1.14 (1.10 - 1.18)	1.05 (0.94 - 1.18)	1.10 (0.98 - 1.24)	0.609
Emergency CS prior to or after start of labor	1.37 (1.33 - 1.41)	1.30 (1.26 - 1.34)	1.34 (1.23 - 1.47)	1.25 (1.14 - 1.37)	0.405
**Birth outcome and post-partum**					
Birth weight, mean grams	-62 (-67 to -57)‡	-49 (-55 to -44)‡	-80 (-98 to -61)‡	-66 (-84 to -48)‡	0.060
Gest. age, mean weeks	-0.31 (-0.33 to -0.29)‡	-0.28 (-0.30 to -0.26)‡	-0.37 (-0.43 to -0.31)‡	-0.33 (-0.39 to -0.27)‡	0.080
Small for gestational age	1.16 (1.10 - 1.24)	1.00 (0.94 - 1.07)	1.26 (1.07 - 1.49)	1.05 (0.89 - 1.24)	0.611
Large for gestational age	1.16 (1.11 - 1.21)	1.10 (1.05 - 1.15)	1.16 (1.00 - 1.35)	1.07 (0.92 - 1.26)	0.782

** Anxiety, depression and asthma from the year before pregnancy until end of pregnancy.*
† Adjusted for country of birth, smoking, cohabitation, maternal education level, BMI, age
‡ P<0.01

Table S2. The association between anxiety/depression during pregnancy* and perinatal characteristics; adjusted logistic and linear full cohort analysis, stratified on birth years 2001-2004 or 2010-2013

	**Current Anxiety/depression 2001-2004 Logistic & Linear Regression Adjusted† OR and beta (95% CI)**	**Current Anxiety/depression 2010-2013 Logistic & Linear Regression Adjusted† OR and beta (95% CI)**
**Pregnancy Characteristics**		
Preeclampsia or Eclampsia	1.07 (0.95 - 1.20)	1.12 (1.06 - 1.19)
Placental abruption	0.93 (0.65 - 1.31)	0.98 (0.79 - 1.23)
**Labor Characteristics**		
Mode of Delivery		
Vaginal non-instrumental delivery	Ref.	Ref.
Elective CS (before start of labor)	1.59 (1.46 - 1.73)	1.59 (1.52 - 1.67)
Vaginal instrumental delivery	1.14 (1.04 - 1.24)	1.18 (1.12 - 1.24)
Emergency CS prior to or after start of labor	1.24 (1.15 - 1.34)	1.33 (1.28 - 1.39)
**Birth outcome and post-partum**		
Birth weight, mean grams	-42 (-55 to -29)‡	-50 (-57 to -43)‡
Gest. age, mean weeks	-0.24 (-0.29 to -0.20)‡	-0.30 (-0.33 to -0.28)‡
Small for gestational age	0.91 (0.78 - 1.06)	1.02 (0.93 - 1.11)
Large for gestational age	1.13 (1.01 - 1.27)	1.12 (1.05 - 1.19)

** Maternal anxiety and/or depression from the year before pregnancy until delivery*
† Adjusted for country of birth, smoking, cohabitation, maternal education level, BMI, age
‡ P <0.001

Table S3. Pregnancies with maternal anxiety/depression diagnose in the year before pregnancy until delivery with or without medication*. Years 2006-2013.

	Current Anxiety/depression with medication		Current Anxiety/depression w/o medication		
	n=11,724		n=6960		
	n(%)		n(%)	Logistic & Linear Regression Crude est (95% CI)	Logistic & Linear Regression Adj† est (95% CI)
**Pregnancy Characteristics**					
Preeclampsia or Eclampsia	626 (5.3)	1.00	292 (4.2)	0.78 (0.67 - 0.90)	0.81 (0.70 - 0.94)
Placental abruption	54 (0.5)	1.00	28 (0.4)	0.87 (0.55 - 1.38)	0.92 (0.57 - 1.47)
**Labor Characteristics**					
Mode of Delivery					
Vaginal non-instrumental delivery	8100 (69.6)	1.00	4819 (69.6)	Ref.	Ref.
Elective CS (before start of labor)	1120 (9.6)	1.00	831 (12.0)	1.25 (1.13 - 1.38)	1.30 (1.17 - 1.43)
Vaginal instrumental delivery	916 (7.8)	1.00	476 (6.8)	0.87 (0.78 - 0.98)	0.89 (0.79 - 1.01)
Emergency CS prior to or after start of labor	1511 (12.9)	1.00	802 (11.5)	0.89 (0.81 - 0.98)	0.94 (0.86 - 1.04)
Missing	77 (0.7)		32 (0.5)		
**Birth outcome and post-partum**					
Birth weight, mean grams	3471	3471	3522	50 (33 to 68)‡	58 (41 to 75)‡
Missing	28 (0.2)		9 (0.1)		
Gest. age, mean weeks	39.2	1.00	38.9	0.25 (0.19 to 0.31)‡	0.25 (0.19 to 0.31)‡
Missing	0 (0.0)		2 (0.0)		
Small for gestational age	287 (2.5)	1.00	153 (2.2)	0.89 (0.73 - 1.09)	0.92 (0.75 - 1.12)
Missing	24 (0.2)		5 (0.1)		
Large for gestational age	496 (4.2)	1.00	304 (4.4)	1.03 (0.89 - 1.20)	1.10 (0.94 - 1.28)
Missing	24 (0.2)		5 (0.1)		

* Anxiety/depression diagnosis in the Swedish National Patient Register. Antidepressant medication dispensed at least twice in the year before pregnancy until birth according to the Swedish Prescribed Drug Register
† Adjusted for country of birth, smoking, cohabitation, maternal education level, BMI, age
‡ P <0.001
